# Is There a Threshold Concentration of Cat Allergen Exposure on Respiratory Symptoms in Adults?

**DOI:** 10.1371/journal.pone.0127457

**Published:** 2015-06-02

**Authors:** Chih-Mei Chen, Elisabeth Thiering, Jan-Paul Zock, Simona Villani, Mario Olivieri, Lars Modig, Deborah Jarvis, Dan Norbäck, Giuseppe Verlato, Joachim Heinrich

**Affiliations:** 1 Paediatric Epidemiology, Institute of Social Medicine, Epidemiology and Health Economics, Charité University Medical Centre Berlin, Berlin, Germany; 2 Helmholtz Zentrum München, German Research Centre for Environmental Health, Institute of Epidemiology, Neuherberg, Germany; 3 Centre for Research in Environmental Epidemiology (CREAL), Barcelona, Spain; 4 Universitat Pompeu Fabra (UPF), Barcelona, Spain; 5 CIBER de Epidemiología y Salud Pública (CIBERESP), Madrid, Spain; 6 Netherlands Institute for Health Services Research (NIVEL), Utrecht, The Netherlands; 7 Department of Health Sciences, University of Pavia, Pavia, Italy; 8 Unit of Occupational Medicine, University Hospital of Verona, Verona, Italy; 9 Occupational and Environmental Medicine, Umeå University, Umeå, Sweden; 10 Respiratory Epidemiology and Public Health Group, Imperial College London, London, United Kingdom; 11 Department of Medical Science, Occupational and Environmental Medicine, Uppsala University, Uppsala, Sweden; 12 Unit of Epidemiology and Medical Statistics, University of Verona, Verona, Italy; University of Texas Health Science Center at San Antonio, UNITED STATES

## Abstract

**Background and Objective:**

Cat allergen concentrations higher than 8 μg/g in settled house dust, have been suggested to provoke exacerbation of allergic respiratory symptoms. However, whether the 8μg/g of indoor cat allergen concentration is indeed the minimal exposure required for triggering the asthma related respiratory symptoms or the development of sensitization has not yet been confirmed. We studied the associations between domestic cat allergen concentrations and allergic symptoms in the European Community Respiratory Health Survey II, with the aim of confirming this suggested threshold.

**Methods:**

Cat allergen concentrations were measured in the mattress dust of 3003 participants from 22 study centres. Levels of specific immunoglobulin E to cat allergens were measured in serum samples using an immunoassay. Information on allergic symptoms, medication use, home environment and smoking was obtained from a face-to-face interview.

**Results:**

Domestic cat allergen concentrations were not associated with allergic/ asthmatic symptoms in the entire study population, nor in the subset sensitized to cat allergen. We also found no association among individuals exposed to concentrations higher than 8 μg/g. However, exposure to medium cat allergen concentrations (0.24-0.63 μg/g) was positively associated with reported asthmatic respiratory symptoms in subjects who have experienced allergic symptoms when near animals.

**Conclusions:**

The proposed 8 μg/g threshold of cat allergen concentrations for the exacerbation of allergic/ respiratory symptoms was not confirmed in a general European adult population. Potential biases attributable to avoidance behaviours and an imprecise exposure assessment cannot be excluded.

## Introduction

Several studies have investigated the effect of cat allergen exposure on respiratory health, but the results are complex and inconclusive [1–4]. Nevertheless, it is clear that patients who experience allergic symptoms when exposed to cat allergens should avoid exposure in order to prevent exacerbations. Studies conducted in the early 90s suggests that cat allergen (Fel d 1) levels measured in the settled dust of a dwelling with a cat usually exceeded 8μg/g and that entering a house with a cat is associated with a rapid onset of allergic symptoms in patients[5]. Therefore, it has been suggested that 8 μg Fel d 1 per gram of dust may be an exposure threshold for cat sensitization and asthma. Levels less than 1μg/g are considered as unlikely to induce sensitization or symptoms [5]. Although these thresholds have not been confirmed in epidemiological studies, 8μg/g has been used as the minimal exposure required for the induction of the development of sensitization and asthma symptoms in studies on children. Furthermore, studies in adult populations use this threshold when assessing exposure-response relationships between cat allergen and existing symptoms or diseases exacerbation [6–9]. In contrast, the effects of exposure to low amounts of airborne cat allergen have been investigated using experimental cat exposure chambers[10]. The results of that study indicated that exposure to airborne Fel d 1 levels less than 0.1μg/m^3^ is sufficient to provoke upper respiratory symptoms and to cause a fall in FEV_1_ of more than 25% in cat sensitized patients [10]. The study also demonstrated that there is only a weak correlation between airborne Fel d 1 and Fel d 1 in settled dust.

As cat allergen is ubiquitous, not owning a cat does not prevent cat allergen exposure [11, 12]. For patients suffering from allergic respiratory symptoms and for susceptible subjects with a high risk of further symptom development, clear guidance on a “safe level” of cat allergen exposure is desirable. In the second European Community Respiratory Health Survey (ECRHS II), cat allergen concentrations were measured in mattress dust samples collected from 22 European study centres in 10 European countries. In a previous investigation, Chinn et al. demonstrated a positive trend between domestic cat allergen concentrations and bronchial hyperresponsiveness to methacholine challenge in sensitized subjects [13]. In the current analysis, we describe the exposure-response relationship between a wide range of cat allergen concentrations and apparent clinical respiratory symptoms. Furthermore, we examine the nature of the association, whether it is linear or whether a threshold indeed exists; as well as the appropriateness of the previously suggested 8μg/g domestic cat allergen concentration as the minimal exposure required for the exacerbation of asthmatic and allergic symptoms.

## Method

### Study design

The European Community Respiratory Health Survey (ECRHS, 1990–1993) was designed as a multicentre cross-sectional study which aimed to estimate variations in the prevalence of asthma, asthma-like symptoms, airway responsiveness, as well as exposures to risk factors for asthma and treatment in European adults [14]. The participants of the survey were sub-divided into a random sample and a symptomatic sample. The symptomatic sample consisted of subjects who reported respiratory symptoms in the ECRHS I screening questionnaire. In 1999–2001, the survey was extended to a multicentre cohort (ECRHS II) [15] which included 29 European centres. The aim was to determine the incidence of and risk factors for the development of allergic disease, atopy and rapid loss of lung function. This article included data from a sub-group of both the random and the symptomatic samples from 22 centres in 10 European countries who participated in the domestic exposure assessment. Ethical approval was obtained from the appropriate institutional or regional ethics committee for each centre, and written consent was obtained from each participant. Ethik-Kommission der Bayerischen Landesärztekammer approved local research. The co-ordination of ECRHS II was supported by the European Commission, as part of Quality of Life program.

### Dust sampling and allergen extraction

Exposure to cat allergen was assessed by analyzing mattress dust samples taken from participants’ mattresses between July 2000 and November 2002. A subset of homes from each center were visited by trained fieldworkers, in random order and covering all seasons. A mattress-area under the bed linen of 1 m^2^ was vacuumed for 2 minutes. Mattress covers or protectors that had been in place for at least 3 months prior to sampling were left on the mattress. Within the next 3 days, samples were frozen at a temperature of -20°C for 24 hours in order to kill mites, and were subsequently stored at room temperature in darkness. In a central laboratory the dust samples were sieved to remove larger particles and to obtain a more homogeneous fine dust for extraction. Extracts, 5% wt/vol, in borate-buffered saline of sieved dust samples were assayed for cat allergen *Felis domesticus* (Fel d1) as well as mite allergen Der p 1, Der f 1, and Der 2 using a mAb ELISA assay (Indoor Biotechnology, Cardiff, United Kingdom). The analytic limit of detection (LOD) was 0.1 μg allergen per gram of dust, with no upper limit. Cat allergen levels below the detection limit were set to 0.05 μg/g. The concentration of Der 1 allergens was the sum of Der p 1 and Der f 1 concentrations. Levels below the detection limit were ignored.

### Blood measurements

Blood samples were collected during the ECRHS II study period and specific immunoglobulin E (IgE) to common allergens were measured. Serum IgE to cat allergen was measured by using the AutoCAP system (Pharmacia, Uppsala, Sweden). Sensitization to cat allergen was considered to be positive if cat-specific IgE concentrations greater than 0.35 kU/L were detected.

### Questionnaire information

Information on the clinical history of asthmatic symptoms, home environment, smoking behaviours, environmental tobacco exposure, and cat ownership was collected during a face-to-face interview. Participants were defined as having asthma using a self reported physician-diagnosis of asthma. The presence of lower respiratory symptoms (wheezing, whistling in the chest, shortness of breath attack during the day at rest, being woken by shortness of breath, being woken by a feeling of chest tightness, and asthma attacks) in the preceding 12 months was ascertained. Allergic upper respiratory symptoms were defined as sneezing (runny or blocked nose) accompanied by itchy or watery eyes in the absence of a cold.

### Statistical analysis

Fel d1 was log transformed as its distribution was skewed. The Kruskal-Wallis rank sum test was performed to determine crude differences in medians between groups. Associations between domestic cat allergen exposure and asthma with lower respiratory symptoms and upper respiratory symptoms were calculated using generalized additive mixed models (gamm) with p-splines with 5 degrees of freedom. Gamm models assess both the linearity and the approximate significance of the associations between Fel d1 exposure and health outcomes. Generalized linear mixed models (glmm) were additionally used to validate the formerly suggested Fel d1 threshold. Variability between the European centres was taken into account by introducing a random effect into the model (14). Models were adjusted for sex, frequency of vacuuming the mattress (never/ less than once per month/ more than once per month), use of mattress-protector, cat ownership, and smoking. In sensitivity analyses, participants currently using asthma medication or steroid nasal spray/ antihistamines against allergy in the past 12 months were excluded. All analyses were performed using R version 2.10.1, with additional packages MASS and mgcv.

## Results

Cat allergen concentrations in settled mattress dust were measured in 3003 subjects’ homes from 22 study centres. The age of the study population ranged from 27 to 56 years and 53% were female. The baseline characteristics of the study population are listed in Table 1. A total of 619 (20.6%) samples contained cat allergen below the lower detection limit. The measured domestic cat allergen concentrations ranged from 0.05 to 123000 μg/g, with a median of 0.37 μg/g and interquartile range (IQR) of 0.12 to 2.64 μg/g. As shown in previous reports [16], the levels varied significantly between study centres (p<0.01). The median level of cat allergen concentration in the mattress dust of cat owners was 94.24 μg/g, with an IQR of 10.83 to 490.80 μg/g. The median cat allergen concentration in homes without a cat was 0.24 μg/g (IQR = 0.05–0.68).

**Table 1:** Baseline characteristics of the study population (subset of ECRHS II).

	n/N (%)
**symptomatic asthma (asthma with lower resp. symptoms)**	353/3003 (12%)
**allergic symptoms near cat/dog/horses**	461/3003 (15%)
**allergic upper resp. symptoms (itchy eyes accompanied by sneezing, runny or blocked nose in absence of a cold)**	709/3003 (24%)
**any lower respiratory symptoms**	1301/3003 (43%)
**reference group** ^**1**^	927/3003 (31%)
**Gender**	
male	1404/3003 (47%)
**Age**	
27-38y	740/3003 (25%)
38-44y	692/3003 (23%)
44-50y	825/3003 (27%)
50-56y	746/3003 (25%)
**sensitized to cat (> 0.35kU/L)**	311/3003 (10%)
**cat ownership**	580/3001 (19%)
**cloth protector or blanket on mattress**	1342/2971 (45%)
**smoking and ETS** ^**2**^	
current smoking	866/2904 (30%)
current ETS^2^	615/2904 (21%)
former smoking and current ETS	532/2904 (18%)
no ETS, no smoking	891/2904 (31%)
**vacuuming of mattress**	
Never (or no vacuum cleaner)	1408/2906 (48%)
Less than once a month	1123/2906 (39%)
More than once a month	375/2906 (13%)

^1^ Never had asthma and no lower respiratory symptoms (wheezing/whistling, tightness in chest, attack of shortness of breath during rest/activity, woken up by an attack of shortness of breath, asthma attack, medication), trouble with breathing, hay fever, runny or blocked nose without a cold, or medicine for breathing (last 12 months)

^2^ exposure to second-hand smoke, smoke at home or during work

**Fig 1:**
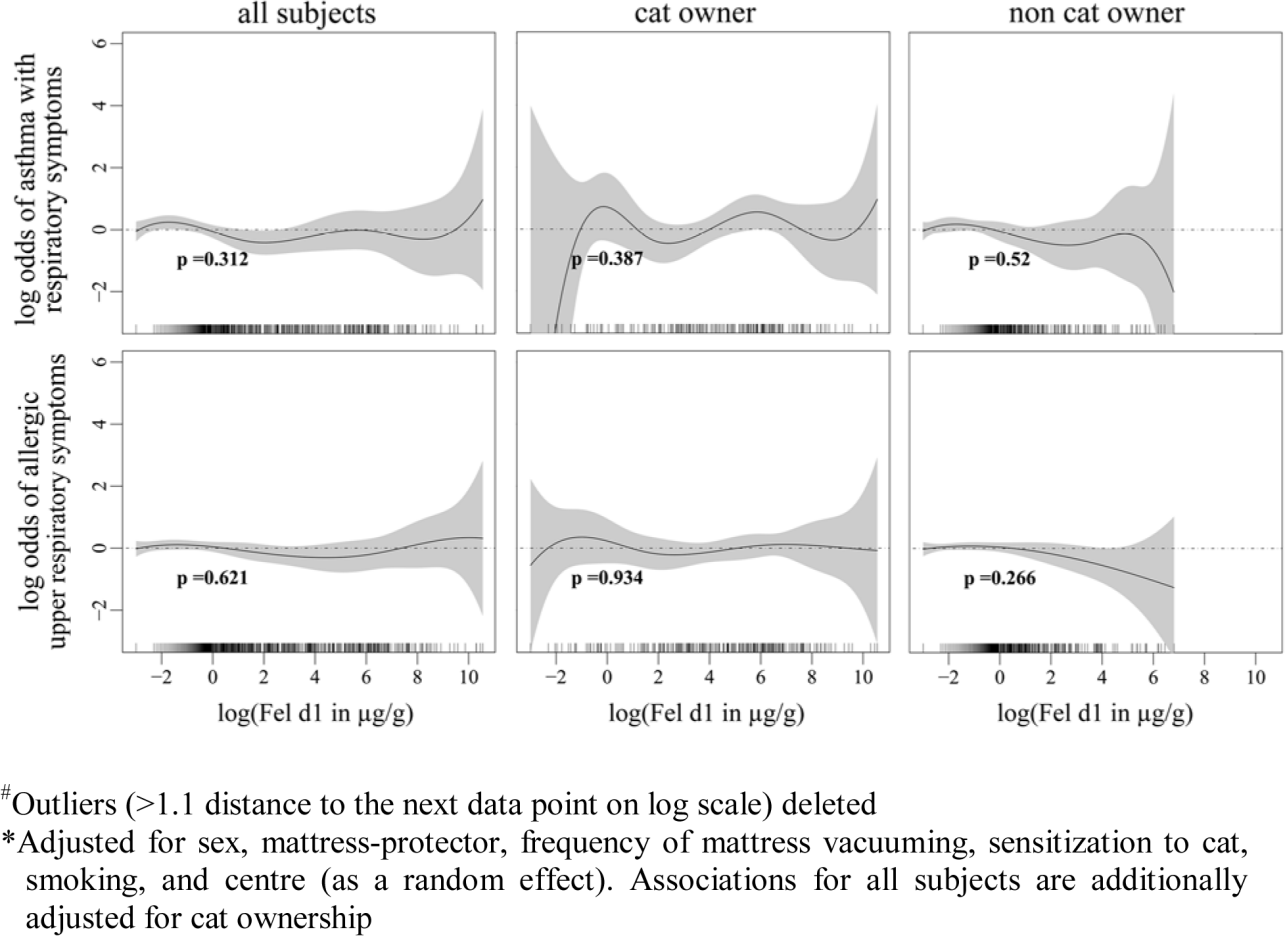
Associations between cat allergen concentrations in mattress dust and asthmatic and allergic respiratory symptoms. Fig 1 shows the associations between log-transformed cat allergen concentrations in mattress dust and asthmatic and allergic respiratory symptoms with approximate significance of the smooth terms

Associations between different levels of domestic cat allergen exposures and reported asthma with current lower respiratory symptoms as well as current allergic upper respiratory symptoms, were assessed using generalized additive models and are presented as smoother plots (Fig 1). Asthma with current lower respiratory symptoms is defined as: ever been diagnosed with asthma by a physician and experienced one or more of the lower respiratory symptoms including wheezing, whistling in the chest, shortness of breath attack during the day at rest, being woken by shortness of breath, being woken by a feeling of chest tightness, and asthma attacks in the previous 12 months. The current allergic upper respiratory symptoms are defined as having experienced sneezing, runny, or blocked nose accompanied by itchy or watery eyes in the absence of a cold in the past 12 months. The associations were analysed in symptomatic subjects versus the reference group, comprised by subjects who had never been diagnosed with asthma or hay fever by a physician, who reported no asthmatic symptoms or trouble with breathing, who were not taking medication for breathing 12 months before the survey and who never had allergic symptoms when in contact with dogs, cats or horses. The smoother plot showed no evidence of a linear exposure-response relationship and no obvious thresholds beyond which exposures to domestic cat allergen increased the odds of reporting asthma with current lower respiratory symptoms and current allergic upper respiratory symptoms. Sixty-four (11%) current cat-owners and 288 (12%) non-cat owners have reported asthma with current respiratory symptoms and one-hundred and thirty one (23%) current cat-owners and 578 (24%) non-cat owners have reported current allergic upper respiratory symptoms. The analysis using a generalized additive model was also performed in the sub-group of cat owners and non-cat owners, and no statistically significant associations between cat allergen exposures and reported symptoms were present.

We specifically tested the proposed threshold of 8μg/g domestic cat allergen concentration by stratifying the analysis into undetectable exposures (<0.1 μg/g), high exposures (> 8μg/g), and 3 groups based on the quartiles of the medium exposure level (0.1–0.23, 0.24–0.63, 0.64–7.9μg/g). Associations between cat allergen exposure and reporting any lower respiratory symptoms in the previous 12 months was assessed amongst subjects who reported symptoms versus the reference group, as well as amongst the sub-groups of those sensitized to cat allergen (serum specific IgE > = 0.35 kU/L) and those who reported ever having allergic or respiratory symptoms when near animals. The prevalence of reporting lower respiratory symptoms in the previous 12 months was not associated with the 8μg/g concentration threshold (Table 2). However, although not statistically significant, the chances of reporting symptoms were slightly increased among those exposed to a medium level of cat allergen (0.24–0.63 μg/g). In a sensitivity analysis in which subjects who reported current use of asthma medication or the use of steroid nasal spray/ antihistamines against allergy in the previous 12 months were excluded, there was a significantly higher chance of experiencing symptoms for subjects exposed to medium levels of cat allergen (0.24–0.63 μg/g) (Table 3), but only among those who had reported previous experience of symptoms when near animals.

**Table 2:** Associations between cat allergen exposures stratified based on the previously defined threshold and asthmatic respiratory symptoms.

Any lower respiratory symptoms v.s. reference group						
μg/g	any lower resp. symp.			sensitized to cat	allergic symptoms near cat/dog/horses	
	n	OR^1^ (CI)	N	OR^1^ (CI)	n	OR^1^ (CI)
undetectable	477	Ref.	228		233	
0.10–0.23	480	1.02 (0.77–1.35)	241	1.00 (0.60–1.66)	270	1.25 (0.78–1.98)
0.24–0.63	412	1.03 (0.76–1.39)	210	1.09 (0.64–1.85)	241	1.47 (0.91–2.38)
0.64–8.0	427	0.90 (0.66–1.22)	232	1.03 (0.61–1.75)	252	1.19 (0.73–1.94)
>8.0	432	0.98 (0.72–1.32)	217	0.97 (0.57–1.64)	228	0.99 (0.60–1.65)

^1^ Adjusted for sex, smoking, mattress-protector, frequency of mattress vacuuming and center (as a random effect)

**Table 3:** Associations between cat allergen exposures stratified based on the previously defined threshold and reported asthmatic respiratory symptoms, excluding subjects who reported current use of asthma medication or the use of steroid nasal spray/ antihistamines for allergy in the past 12 months.

Any lower respiratory symptoms v.s. reference group						
μg/g	any lower resp. symp.		sensitized to cat		allergic symptoms near cat/dog/horses	
		OR^1^ (CI)		OR^1^ (CI)		OR^1^ (CI)
undetectable	409		208		205	
0.10–0.23	400	0.93 (0.69–1.26)	214	0.68 (0.33–1.38)	230	1.25 (0.66–2.36)
0.24–0.63	351	1.09 (0.80–1.50)	186	1.08 (0.55–2.12)	207	**2.13 (1.15–3.97)**
0.64–8.0	361	0.82 (0.60–1.14)	206	0.92 (0.46–1.84)	219	1.33 (0.70–2.56)
>8.0	370	0.97 (0.70–1.33)	191	0.84 (0.42–1.70)	191	0.83 (0.41–1.69)

^1^ Adjusted for sex, smoking, frequency of mattress-protector, mattress vacuuming and centre (as random effect)

**Fig 2:**
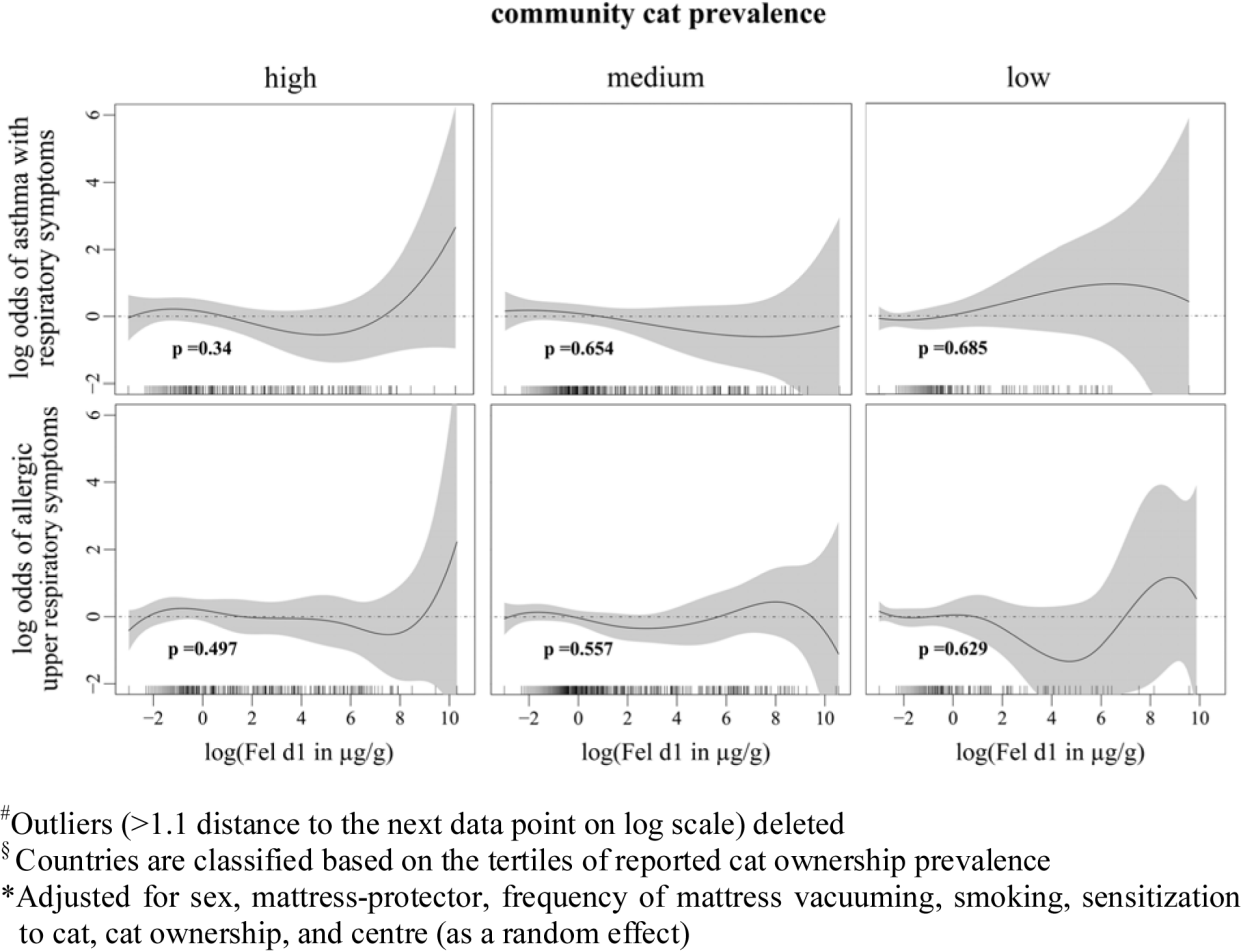
Associations between domestic cat allergen exposure and asthmatic and allergic respiratory symptoms stratified by community cat prevalence. Fig 2 shows the associations between log-transformed cat allergen concentrations in mattress dust and asthmatic and allergic respiratory symptoms with approximate significance of the smooth terms, stratified by community cat prevalence

**Fig 3:**
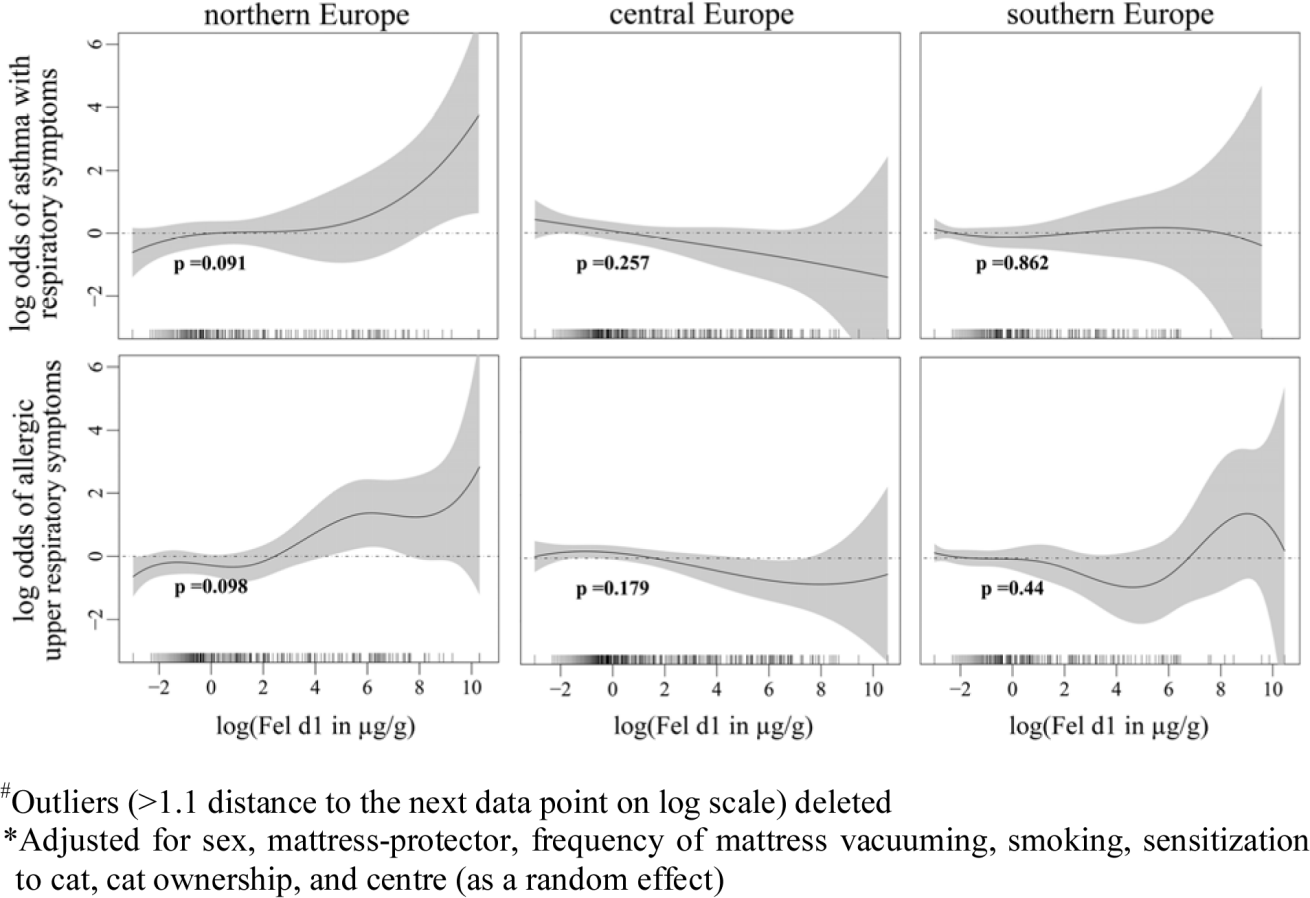
Associations between domestic cat allergen exposure and asthmatic and allergic respiratory symptoms stratified by geographical location. Fig 3 shows the associations between log-transformed cat allergen concentrations in mattress dust and asthmatic and allergic respiratory symptoms with approximate significance of the smooth terms, stratified by geographical location

Cat allergen is ubiquitous [11, 12]; living in a community with high cat ownership also means higher levels of cat allergen exposure and it may therefore mask the effect of domestic cat allergen exposure. Stratifying our subjects by the community prevalence of cat ownership, we found no association between the domestic cat allergen exposure and odds of reporting asthma with lower respiratory symptoms and allergic upper respiratory symptoms (Fig 2). Geographical locations are typically associated with the mixtures of various allergen exposures; for example, the quantity of domestic allergen house dust mite is low in northern Europe and high in southern Europe due to the differences in climate [17]. Exposures to certain mixtures of allergens may also facilitate or inhibit the effect of domestic cat allergen exposures on symptom presentations. Stratified analyses based on the geographical location of our study subjects showed a positive but not statistically significant exposure-response relationship between domestic cat allergen exposure and reported asthma with lower respiratory symptoms in Northern European countries (Fig 3).

## Discussion

We were unable to show evidence of a dose-response association between domestic cat allergen concentrations and respiratory symptoms in adults living in a wide range of European communities. For adults who had previously experienced allergic or respiratory symptoms when near animals, exposure to medium levels of domestic cat allergen (0.24–0.63 μg/g) was associated with increased lower respiratory symptoms. We found no evidence to support the existence of a threshold of domestic cat allergen concentration (8 μg/g and 1 μg/g) beyond which symptoms are exacerbated in adults sensitized to cat allergen.

Cat allergen levels varied significantly between homes with and without a cat. In the present study, 50% of non-cat owners were exposed to cat allergen concentrations lower than 0.24 μg/g. This low exposure level in settled house dust is unlikely to cause irritations. Previous investigations of the ECRHS data showed evidence of selective avoidance (i.e. not purchasing a cat) after asthma or allergy development [18]. Cat owners who are sensitized to cat allergen or who developed allergic symptoms may reduce their exposure by not allowing their cat in the bedroom or eventually giving the cat away. In our study, 64% of the subjects who gave their cat away between the two ECRHS surveys allowed their cat in their bedrooms. Comparatively, among those who still had their cat at the time of the second survey, 94% allowed their cat in their bedroom. Furthermore, the risk of new-onset cat sensitization was slightly increased among the subjects who gave their cat away between the two surveys versus those without a cat at both surveys. [19]. These observations support the possibility of a reverse causation; allergic symptoms towards cats are associated with a decrease in allergen concentrations at home. There may nevertheless be subjects who refuse to take avoidance actions, regardless of their symptoms. However, we can assume this subgroup is small and therefore cannot be properly studied in the current analysis. Additionally, sensitized subjects may not experience any symptoms despite their exposures to cat allergen, possibly due to the use of asthma medication, nasal steroid spray, or antihistamines for allergy. Our results show that after excluding those who reported medication use, exposure to a medium level of domestic cat allergen (0.24–0.63 μg/g) was associated with increased lower respiratory symptoms in subjects who experience allergic or respiratory symptoms when near animals. In this particularly sensitive group, relatively low doses of exposure to animal allergens may trigger symptoms. However, it is not possible to completely avoid the ubiquitous cat allergens at home, and the subjects may not yet realize that cat allergen in their cat-free homes could be associated with their symptoms. A limitation of the current study is that we could not take acute respiratory symptoms into account. The outcomes used in the current analysis were defined based on symptoms in the past 12 months. Subjects who have strong adverse health effects when exposed to cat may avoid owning a cat at home but may experience acute symptoms when exposed to cat allergen in other places.

In a previous analysis of ECRHS data, a positive association between exposures to cat allergen in mattress dust and bronchial hyperresponsiveness to methacholine challenge was observed in atopic subjects[13]; however, we did not observe any association between domestic cat allergen exposure and reported symptoms in our current analysis. In another double-blind, placebo-controlled allergen provocation study conducted in France, results showed that exposure to repeated low doses of cat allergen through inhalation, activates inflammation cell eosinophil and increases non-specific bronchial hyperresponsiveness but not clinical symptoms, in non-symptomatic, cat-sensitized asthmatics who were free of medication for at least one month before the experiment[20]. In this allergen provocation study, the subjects in the study arm received consecutive low dosages of cat allergen exposure, ranging from 2.1 to 7.0 ng, which was one fifth of the corresponding cumulative dose of Fel d 1 that induced a positive response in the conventional bronchial challenge test carried out for each subject at baseline. Repeated exposures to sub-clinical doses of allergen can be viewed as a simulation of the natural environmental allergen exposure scenario, and the variation of the tolerance levels between subjects resembles the variation of the measured cat allergen concentrations in domestic mattress dust. The results of the allergen provocation study, as well as the observations from both the current study and the previous analysis by Chinn et al. using the ECRHS data, suggest that atopic subjects may tolerate a relatively low exposure to domestic cat allergen, as the exposure dosage was not sufficient to induce clinical manifestations, but was sufficient to cause sub-clinical allergic inflammation in the airways and a trend toward greater bronchial responsiveness with increasing exposure to cat allergen. These results also indicate that long-term, relatively low-dose domestic cat allergen exposure, may lead to adverse effects on respiratory health in atopic subjects, even without causing perceptible symptoms.

In our analysis, the exposure-response relationships between domestic cat allergen and reported current respiratory symptoms varied by study centers, as reported in the previous analysis of the ECRHS data [3]. When grouping the study centers based on geographical location, a positive but not statistically significant effect of cat allergen exposure on current asthma-like symptoms among asthmatics was observed in centers from northern European countries, where indoor mite allergen levels are significantly lower [17]. It is thus possible that in other countries, the health effects resulting from cat allergen exposure are masked by the effect of exposures to high domestic mite concentrations. However, additionally adjusting for the level of house dust mite Der 1 concentrations in mattress dust did not alter the health effect estimates for cat allergen exposure; therefore additional exposures to mite allergen cannot explain the observed null association in our study. In the previous study using the ECRHS data, it was suggested by the authors that any effect of domestic cat allergen exposure might be modulated by the community prevalence of cats [21]. As cat allergen is more dominant in northern European countries, its effect may also be more influential in these areas. On the other hand, it is difficult to disentangle the observed effects using only community cat allergen exposures, especially in adult populations, as exposures to other respiratory irritants such as secondhand tobacco smoke, outdoor air pollution, or occupational exposure to chemical agents, may also affect an individual’s susceptibility to cat allergen exposure [16, 22].

Cat allergen is ubiquitous [11, 12]. Adults may be exposed to cat allergen at work, in public buildings, or in their relatives’ or friends’ homes. Domestic cat allergen exposure can thus only account for a certain proportion of overall exposure and this proportion is likely to vary between individuals. Although only considering cat allergen concentrations in mattress dust seems to ignore allergen sources from other locations in the home, such as the sofa and floor, cat allergen concentrations in different locations in one household have been shown to be highly correlated with cat allergen concentrations in mattress dust (ρ = 0.8) [23]. Furthermore, mattress dust is more homogeneous than floor dust. Therefore, the cat allergen concentration in mattress dust is a good indicator for domestic cat allergen exposure. A recent study also demonstrated that when examining the relationship between cat allergen exposure and asthma severity, cat allergen concentrations measured from dust samples are more useful than samples collected via nasal air samplers [24].

## Conclusion and Interpretation

Evidence to support the previously proposed minimum cat allergen concentration in settled house dust of 8μg/g, as a threshold beyond which respiratory symptoms are exacerbated in adults sensitized to cat allergen, was not observed in this community survey. However, the observed null exposure-response associations may be partly due to avoidance behaviours or an imprecise exposure assessment. Furthermore, as the current study population is restricted to adults, the observed results may not be generalizable to children.
